# Conservative treatment of a psoas hematoma revealed by a lower limb palsy

**DOI:** 10.11604/pamj.2017.28.138.9930

**Published:** 2017-10-13

**Authors:** Zidouh Saad, Belkouch Ahmed, Rafai Mostafa, Bakkali Hicham, Belyamani Lahcen

**Affiliations:** 1 Emergency Department, Military Hospital, Rabat, Morocco

**Keywords:** Palsy, hematoma, conservative treatment

## Abstract

The psoas hematoma is a rare complication of the anticoagulation therapy. It cause abdominal or lumbar pain, muscle dysfunction and sometimes nerve palsy. The optimal treatment is not well established, surgery or conservative treatment? We report here a case of psoas hematoma revealed by a lower limb palsy.

## Introduction

A palsy of the lower limb can be caused by the lumbar disc prolapse, but if the patient is under anticoagulation therapy, the psoas hematoma is a diagnosis not to miss even if it’s rare.

## Patient and observation

**Figure 1:**
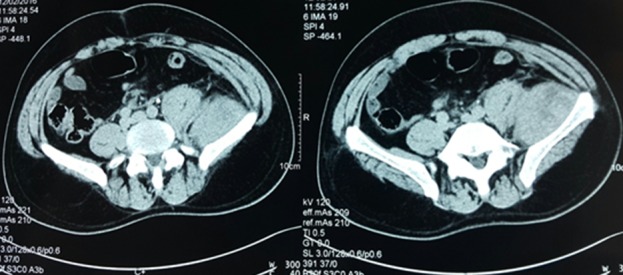
The left psoas thickening due to hematoma

In January 2016, a 48-year-old man presented with a 02-day history of pain and progressive spontaneous swelling of the left thigh. He had progressive limitation of the homolateral knee. He was receiving oral anticoagulation therapy for cerebral vascular accident since 2013 and valvular replacement since 2014. There was no visible haematoma in the lower limb. Neurological examination showed a complete functional deficit of all muscles dependent on the right lumbar plexus (femoral and obturator nerves), with hypoesthesia and abolition of the ipsilateral knee reflex. Computed tomography of the pelvis showed a massive haematoma, (7x4 cm) of the psoas and the iliac muscle (Figure 1). Blood tests showed hypocoagulation (Quick value 9%; PTT: 159/29; INR: 9,27) and anaemia (Hb: 9,4 g/dL; Ht: 29,6%). A fresh frozen plasma was transfused because the PPSB wasn’t available. Anticoagulation was discontinued and the patient takes 10 mg of phytomenadione. Haemoglobin levels remained stable for 4 days, thereby excluding active bleeding. However, complete recovery functional deficit of the lumbar plexus musculature were obtained after 4 weeks. Considering the blood condition of the patient and the manifested neurological injury, drainage of the haematoma was rejected, because of the increased risk of bleeding complications and mortality. Conservative management was continued. Electromyographs showed complete recovery and no signs of denervation or other axonal lesion. At the three-month follow-up, the patient was entirely asymptomatic.

## Discussion

Haematomas of the iliac psoas muscle are rare: 0,1 à 0,6%. Risk factors for spontaneous retroperitoneal hematoma are anticoagulation therapy, the elderly and hemodialysis. It’s a disease with a high rate of mortality: 30% [1]. Several reports referred to traumatic iliac psoas haematomas due to blunt trauma or rupture of the muscle itself [2], but the majority of the patients so far published suffered a spontaneous haemorrhage. Most of the patients were on acute or chronic anticoagulant therapy, specifically heparin [3-5], and it appears that with increased use of anticoagulant agents, spontaneous haemorrhage within the iliac psoas muscle has become a more frequent clinical problem [3]. The most common cause of the lumbosacral plexus injury is a haematoma at the iliacus compressing the femoral nerve, followed by a haematoma at the psoas compressing the femoral and obturator nerves and causing diffuse injury to the lumbar plexus [6]. Patients usually consult for pain at the groin with progressive difficulty in walking several days after a low-energy trauma. In most cases, the first neurological symptom detected is hypoesthesia in the suprapatellar area [7]. Magnetic resonance imaging has high sensitivity for the detection of small haematomas, but is not widely available. Computed tomography is the most common imaging tool [6, 8-10]. Treatment ranges from drainage of the haematoma to monitoring of the neurological injury. Surgery is recommended for trauma patients, large haematomas, and those with progressive neurological impairment, whereas conservative treatment is reserved for haemophilia patients and those with coagulation disorders [8, 10]. Percutaneous drainage should be attempted first before surgical decompression, despite the difficulty of draining intramuscular haematomas. This enables faster recovery and avoids sequelae [2, 9]. Nonetheless, the natural evolution of the injury is spontaneous resolution. Our patient was receiving anticoagulation for a cerebral and heart condition. He had complete injury to the lumbar plexus for 2 days after a spontaneous swelling of the tigh. Surgical drainage of the haematoma at the psoas was rejected because of the increased risk of bleeding complications and mortality. We choose to stop the anticoagulation for 72 h. Periodic monitoring of the neurological injury was thus performed. The patient described in this article had been treated conservatively and was entirely asymptomatic thereafter.

## Conclusion

Patients under anticoagulation therapy have a significant risk of bleeding and any new symptom should be taken into account in search of a hidden hemorrhage. A conservative treatment in this situation is the best choice to avoid more complications.

## Competing interests

The authors declare no competing interest.
